# Virtual follow-up and care for patients with cardiac electronic implantable devices: protocol for a systematic review

**DOI:** 10.1186/s13643-020-01406-6

**Published:** 2020-06-27

**Authors:** Shannon E. Kelly, Tammy J. Clifford, Doug Coyle, Janet Martin, Vivian Welch, Becky Skidmore, David Birnie, Ratika Parkash, Anthony S. L. Tang, George A. Wells

**Affiliations:** 1 School of Epidemiology and Public Health, University of Ottawa, 101 - 600 Peter Morand Crescent, Ottawa, Ontario K1G 5Z3 Canada; 2 Department of Epidemiology and Biostatistics, Western University, London, Ontario Canada; 3 Independent Information Specialist, Ottawa, Ontario Canada; 4 University of Ottawa Heart Institute, Ottawa, Ontario Canada; 5 Division of Cardiology, Dalhousie University, Halifax, Nova Scotia Canada; 6 Schulich School of Medicine and Dentistry, Western University, London, Ontario Canada

**Keywords:** Remote monitoring, Virtual follow-up, Virtual care, eHealth, Distance, Implantable device, Medical device, Models of care, Distance factors, Cardiac implantable electronic device, ICD, Pacemaker, CRT

## Abstract

**Background:**

Capacity to deliver outpatient care for patients with cardiac implantable electronic devices (CIEDs) may soon be outweighed by need. This systematic review aims to investigate the comparative effectiveness, safety, and cost for virtual or remote clinic interventions for patients with CIEDs and explores how outcomes may be influenced by patient or system factors in-depth.

**Methods:**

We will perform a systematic literature search in MEDLINE, Embase, PsycINFO, CINAHL, Proquest Dissertations & Theses, other EBM Reviews, and trial registry databases. Two authors will independently screen titles and abstracts for eligibility. We will include randomized and non-randomized controlled trials, quasi-randomized and experimental studies, cohort, and case-control studies. Study populations of interest are individuals with a CIED (pacemaker, ICD, CRT). Eligibility will be restricted to virtual or remote follow-up or care interventions compared to any other approach. The co-primary outcomes of interest are mortality and patient satisfaction. Secondary outcomes include clinical effectiveness (e.g., ICD shock, time-to-detection of medical event, hospitalizations), safety (e.g., serious or device-related adverse events), device efficacy (e.g., transmissions, malfunctions), costs, workflow (e.g., resources, process outcomes, time-saved), and patient reported (e.g., burden, quality of life). Data will be extracted by one author and checked by a second using a standardized template. We will use published frameworks to capture data relevant to intervention effects that may be influenced by intervention definition or complexity, context and setting, or in socially disadvantaged populations. Detailed descriptive results will be presented for all included studies and outcomes, and where feasible, synthesized using meta-analysis. Risk of bias will be assessed by two review authors independently using Cochrane Risk of Bias tools. Certainty of evidence will be assessed using the GRADE approach.

**Discussion:**

Increases in number of CIEDs implanted, combined with an aging population and finite health resource allocations at the system-level may lead to increased reliance on virtual follow-up or care models in the future. These models must prioritize consistent, equitable, and timely care as a priority. Results from this systematic review will provide important insight into the potential contextual factors which moderate or mediate the effectiveness, safety, and cost of virtual follow-up or care models for patients.

**Systematic review registration:**

PROSPERO registration number CRD42020145210

## Background

It is standard practice for patients with cardiovascular electronic devices (CIEDs) to visit specialty device outpatient clinics for follow-up; however, demands on these services may begin to outweigh available resources as populations age and more devices are being implanted [1, 2]. Routine follow-up appointments can burden both patients and the outpatient clinics who manage follow-up care. Patients and their caregivers often travel long distances to attend appointments, and pacing clinics often struggle to manage the volume of in-clinic visits while maintaining optimal care for patients. Routine in-clinic visits contribute to delays in access to appointment times for patients with greater clinical need or urgency. Over the last decade, advances in telecommunications and device technology have led to innovations in arrhythmia care [1]. Virtual outpatient visits (also known as *remote interrogation or monitoring*) are now used in many regions in a blended model combined with traditional in-clinic visits or as a means to follow patients more closely when they are subject to device recalls, alerts, or are close to requiring replacement of the device, leads, or battery [2]. In some jurisdictions, these virtual follow-up visits, alternated every 6 to 12 months with outpatient device clinic visits, for CIEDs are now considered standard of care [2, 3].

Virtual models of follow-up for pacemakers, implantable cardioverter defibrillators (ICDs), and cardiac resynchronization therapy (CRT) offer patients the convenience of staying in their own home while still obtaining quality care, a benefit that may especially be impactful to those living in rural or remote areas. Use of virtual surveillance and follow-up “visits” is often described using the term “remote monitoring.” The value of virtual models of follow-up lies in the potential to monitor patients and their devices as effectively and safely as if they had attended an in-person appointment. Several randomized clinical studies have demonstrated that virtual follow-up is a broadly safe and beneficial alternative to in-person outpatient clinic visits in adult populations living with CIEDs, although there is less clinical evidence for pacemaker and CRT populations [4–8]. There is also interest in exploring the feasibility of eliminating routine in-clinic follow-up visits in favor of only virtual follow-up and surveillance. The RPM CIED randomized controlled trial currently underway in Canada is evaluating a virtual-only model for CRT, ICD, and pacemakers alongside a user-driver clinical application [9]. Likewise, researchers are studying if more complete virtual care (i.e., including some kind of virtual therapeutic intervention like device reprogramming or automatic calibration of devices using artificial intelligence) for patients can be implemented in patients with adequate battery longevity who are otherwise clinically stable [10–12]. This type of care intervention may also be referred to as “remote patient management” [12] and is not being used as part of standard care yet.

The umbrella phrase “remote monitoring” is used in both research and practice to describe the technology-enabled process where device or patient data and/or diagnostics are collected via passive remote device interrogations and automated transmission of active pre-specified alerts related to device functionality and clinical events are returned to a receptor device clinic. Although a well-recognized term in the clinical realm, it conceptually oversimplifies a clinical intervention which is quite complex [13]. Complex health interventions are usually defined as such because they are (a) multicomponent (i.e., comprised of multiple components that interact in potentially synergistic or discordant ways), (b) non-linear (they may not follow a simple causal pathway to bring about their effects), and (c) context-dependent (although not standardized, they may work best when tailored to local contexts) [14]. All three seem to apply directly to virtual follow-up or care for CIEDs, yet these nuances are often completely overlooked or undocumented in existing research. With virtual follow-up, complexity (1) comes from several interacting components (i.e., patient, device, transmission modality, software, user interfaces, clinic, education, and training), (2) involves groups or individuals at different organizational levels (i.e., patient, vendor, device clinic, institution), (3) has multiple and variable outcomes (e.g., related to device, patient, process, cost, equity), and (4) permits a certain degree of flexibility or tailoring [14].

Clinicians who support the use of virtual follow-up for patients with CIEDs have noted that outside of the research environment, there are issues with the delivery of timely, uniform, and efficient arrhythmia care in practice across health jurisdictions, and uptake is not optimal [15, 16]. It is unclear if the health and system benefits identified in individual research studies (e.g., shorter time to detection and treatment of medical and device-related events, fewer clinic visits [17, 18]) involving virtual follow-up for CIEDs are being realized in practice, and evidence syntheses have not found significant effects on cardiovascular or all-cause mortality, stroke, hospitalization when randomized studies are pooled. Results from these studies also demonstrate wide variation in both patient and device clinic setting along with intervention delivery characteristics related to how remote monitoring is offered to patients, the timing and frequency of follow-up, and data transmission and review which are not investigated further [18]. Recent surveys of both patients and device clinics in Canada have found that virtual follow-up falling under the “remote monitoring” catch-all is extremely heterogeneous in terms of who receives or is offered virtual follow-up; who, when, and how it is delivered; and how resulting data transmissions or alerts are handled [16].

Consequently, it seems overly simple to conclude that virtual follow-up for CIEDs is effective and safe based on an average effect across varying populations and delivery approaches. A lack of understanding about why or how complex virtual interventions work may lead to difficulties in quality improvement, implementation, adaptation, and scaling up of comparable interventions. We are currently unable to judge whether components within these approaches are effective and ineffective (or potentially unsafe), how approaches might be modified by contextual factors or setting, or if there are actual gains for patients in terms of increased accessibility to services and reach (e.g., ability to offer care to patients in rural or remote areas). Existing reviews fail to consider non-randomized or observational study designs which may offer important contextual and organization of care details that offer potential insight and compliment randomized evidence. To scale, improve, and expand virtual follow-up or care offerings across often large and diverse populations and care settings, we need to better understand how context and different intervention components influence outcomes. The present systematic review protocol outlines our approach to comprehensively review the evidence for the comparative clinical effectiveness, safety, and cost-effectiveness of virtual follow-up or care models for patients with CIEDs while considering the complexity of the intervention and the system in which it interacts, and the impact of context and setting.

## Methods

This protocol was written a priori and will be followed throughout the study process. Any deviations from this protocol will be disclosed in all related publications, and the PROSPERO record will be updated accordingly (CRD42020145210). All research questions will be addressed by a de novo systematic review of published clinical evidence. This systematic review protocol has been developed using developed methodological guidance for systematic reviews of complex health interventions [19–26] and considers guidance from the Preferred Reporting Items for Systematic Review and Meta-Analysis–Protocols (PRISMA-P) statement **(**Online Supplement 1) [27].

### Research questions

The systematic review will address the primary research question: *What are the comparative clinical effectiveness, safety, and cost of virtual follow-up or care models for patients with CIEDs?*

Several subsets of the research question will be addressed to additionally capture the complexity of virtual follow-up or care and the context in which it is delivered:

1. What is virtual follow-up or care for CIEDs, and how is it defined?
2. How do the components of virtual follow-up/care interventions work alone or in combination to produce effects?
3. How does context affect the effects of the intervention or cost?
4. How do effects or cost change over time, and what explains change in the effects of the intervention over time?
5. What is the distribution of effects across socially disadvantaged populations [28]?

A qualitative evidence synthesis of patient and health care provider perspectives and care experiences, and implementation barriers is currently underway to supplement available evidence related to the research questions above (PROSPERO submission pending).

### Literature search strategy

An experienced information specialist will design the literature search in consultation with the research team. The electronic search strategy will undergo peer-review by a second, independent information specialist using the Peer Review of Electronic Search Strategies (PRESS) Guideline Statement [29] prior to execution. The complete proposed search strategy is presented in Online Supplement 2.

Information will be identified by searching the following bibliographic databases: Using the OVID platform, Ovid MEDLINE®, including Epub Ahead of Print and In-Process & Other Non-Indexed Citations, Embase Classic + Embase, PsycINFO, and the following EBM Reviews databases: Cochrane Central Register of Controlled Trials, Cochrane Database of Systematic Reviews, Database of Abstracts of Reviews of Effects, Health Technology Assessment, and the NHS Economic Evaluation Database. We will also search CINAHL (EBSCO platform) and Proquest Dissertations & Theses Global.

An initial search strategy was designed and piloted between December 29, 2018, and January 6, 2019. The proposed strategy was run on June 26, 2019, to test for volume. The searches will be conducted in two parts. The main search will apply research design filters, and the second supplemental search will utilize an extensive qualitative filter. Qualitative and case studies will be the focus of the qualitative synthesis and will be reported separately. The core concepts in both parts will be comparable (i.e., remote monitoring and implantable cardiac devices). We will use a combination of controlled vocabulary (e.g., “Remote Consultation”, “Defibrillators, Implantable”, “Cardiac Electrophysiology”) and key words (e.g., telemonitor, pacemaker, CIED) for the concepts in all searches. We will remove animal-only citations and news items where possible from the results. We plan on searching for relevant studies using ClinicalTrials.gov and the International Clinical Trials Registry Platform (ICTRP) Search Portal. Searches will be limited by date to records available after January 1, 2000 (to coincide with the first regulatory approvals of wireless, remote monitoring systems by Biotronik), and not limited in any other way (e.g., by language or publication status). Regular search updates will be performed on databases that do not provide alert services.

### Study eligibility

The eligibility criteria relevant to addressing the research questions can be found in Table 1.

**Table 1:** Eligibility criteria for the research questions

**Population**	
Adults or children (any gender) with a CIED (pacemaker, ICD, CRT). Device may be de novo, existing, or in a patient undergoing a pulse generator change that now has virtual follow-up or care capabilities. Exclusions: • Patients with implantable loop recorders.	
**Intervention**	
**Virtual follow-up** may also be referred to as remote monitoring. In this study we broadly include the collection of device or patient data and/or diagnostics via passive remote device interrogations and the automated transmission of active pre-specified alerts related to device functionality and clinical events. This involves a one-way transmission of data from the patient in their outpatient setting to a receptor device or specialty clinic. Here, the patient alternates virtual follow-up from home with in-person device clinic visits (6 month intervals for ICDs/CRT, 12 month intervals for pacemakers). This can be utilized for any CIED (pacemakers, ICDs, or CRTs) which have the capability. Virtual follow-up is most often suited for patients with stable device function and adequate battery longevity after at least one in-person post-surgical follow-up visit. **Virtual care** may also be referred to as remote patient management and involves therapeutic intervention on the patient’s implanted CIED from a distance using available technology (e.g., remotely re-programming device thresholds, automated recalibration of device settings using machine-learning algorithms). This involves two-way interaction and is informed by virtual follow-up through transmission of data from patient in their outpatient (or non-device clinic) setting to a receptor device clinic and then related care or action involving some kind of therapeutic adjustment from a physician (or qualified individual) at the receptor device clinic back to the patient’s device. This eliminates the need for travel to a specialty device clinic, with exceptions for changes in medical status, battery replacement, or re-implant. Follow-up remains the same from home but patient may be required to travel from home to a local provider for virtual care intervention. This approach may also be accompanied by additional interventions aimed at patient self-efficacy and empowerment (e.g., providing a patient with their own data). Any virtual therapeutic intervention for CIED patients is considered in-scope for this review. Exclusions: • Biometric, non-device-related patient data (e.g., blood pressure, weight) follow-up via telecardiology.	
**Comparators**	
Any (standard care, in-clinic care, transtelephonic monitoring, a different virtual follow-up, or care model^a^)	
**Outcomes**^b^	
**Primary outcomes:** mortality (all), patient satisfaction with care **Secondary outcomes:** **Clinical effectiveness:** Cardiovascular mortality, stroke, ICD shocks (total, appropriate, inappropriate), arrhythmias (pacemaker patients only), time to detection of medical events (time to detection of ventricular arrhythmias, time to detection of atrial fibrillation, time to initiation of anti-coagulation in patients with new device-detected atrial fibrillation, time to detection of device infection, time to detection of device malfunction [including lead malfunction], time from detected medical event to action/decision, time to detection of suboptimal biventricular pacing (defined as < 90% in CRT patients only)), worsening NYHA functional class, response to CRT, total clinic visits (total, scheduled, unscheduled), emergency department visits, hospitalizations (all, device-related, for heart failure, or of cardiovascular cause), length of hospital stay, patient compliance, patient adherence. **Patient-reported or important:** Activities of daily living, burden, self-efficacy, quality of life, empowerment **Device efficacy:** system set-up (attempts, success, failure), transmissions (total, unsuccessful, successful), malfunctions with system, alerts, or data **Safety:** adverse events attributable to intervention, serious adverse events **Costs:** any cost or resource use data identified will be of interest (related to the patient, clinic, or system). These must be economic elements, expressed as quantifiable outcomes or change in outcomes. **Workflow/operational:** staff/physician resources or time, time per “appointment,” process efficiencies, use of time for education or technical training, proportion of clinic patients using virtual follow-up or care, telephone calls made (by type if reported). **Other:** Study withdrawals, group crossovers, composite endpoints involving any of the above outcomes. Equity in terms of geographic/socio-economic access or reach, impact to PROGRESS+ factors.	
**Study design**	
Randomized controlled trials, non-randomized controlled trials, quasi-randomized studies^c^, cohort studies (prospective, retrospective, historical)^d^, case-control studies, quasi-experimental studies. Studies may be from peer-reviewed publications, trial registry records, conference abstracts, letters, presentations, or thesis/dissertation documents. Protocols and trial registry records that meet eligibility criteria will be included, investigated for publication bias, and will be documented or summarized as indicators of “in progress” research. Exclusions: Case reports, case series, review articles, cross-sectional studies, surveys, qualitative or interview/focus group studies, editorials, letters, and commentaries.	
**Context/setting**	
Any setting or context. All outcomes will be explored for meaningful differences measured by contextual factors, or those related to the setting of the device clinic or patient. Differences in PROGRESS-PLUS factors will be of interest, as well as any specified stratified reporting of findings, or evidence of moderating or mediating effects of context or setting.	
**Time frame**	
January 1, 2000, to present.	
**Language**	
No limitations.	

*CIED* cardiovascular implantable electronic device, *ICD* implantable cardioverter defibrillator, *CRT* cardiac resynchronization therapy

^a^“A different virtual follow-up or care model” means any virtual follow-up or care model compared with any other virtual follow-up or care model (i.e., virtual follow-up or care models compared with each other)

^b^All outcomes are considered to be important based on the scoping exercises that informed this review and involved consultation with more than one clinical expert in cardiology and electrophysiology

^c^A quasi-experimental design is an empirical interventional study used to associate outcomes of an intervention on a group of sampled participants without random assignment. For example, pretest-posttest, and interrupted time-series designs

^d^Cohort studies are defined as studies in which a defined group of participants (the cohort) are sampled on the basis of exposure (interventions received) and in which subsequent outcomes and the association with different interventions received are assessed in a follow-up period of time. This is differentiated from case series studies, in which observations are made on a series of participants, usually all receiving the same intervention and who are followed over a defined time to examine subsequent outcomes, without a control group

The primary outcome will be all-cause mortality as this addresses both effectiveness and safety and because prevention of heart rhythm problems causing death is the ultimate treatment goal for any CIED. The co-primary outcome will be patient satisfaction (using any scale, instrument, or tool), as this outcome represents a patient-oriented care priority and may indicate willingness to continue with virtual follow-up or care. Studies will be included if they meet the selection criteria. No studies will be excluded based on the outcomes reported. In cases where studies report mixed populations (i.e., the study is comprised of individuals who do and do not meet the eligibility criteria), we will include studies that report results that apply to the population of interest separately. Where studies do not report results for the mixed population separately, we will include studies where the majority of the mixed population participants meet the eligibility criteria.

### Literature screening and selection

Two independent reviewers will screen the titles and abstracts of all citations retrieved against the eligibility criteria (see Table 1). A citation will not be excluded during screening at the title and abstract stage unless both reviewers agree. All citations deemed by the reviewers to be potentially relevant (or unclear) at the title and abstract stage will be retrieved as full-text articles for a second level of screening by two independent review authors. Discrepancies will be resolved through discussion or consultation with a third reviewer if necessary. We will use standardized forms for article screening and selection set up within DistillerSR. The full literature screening and selection process will be documented and presented in a PRISMA flow diagram [30].

### Data extraction

Information will be extracted from all included studies, including bibliographic information details pertinent to study characteristics (e.g., name of first author, publication year, publication title, country, funding sources), research methodology (e.g., study design, aim or objectives, inclusion and exclusion criteria, recruitment method, primary and secondary outcomes, subgroup analyses of interest), population (e.g., number of patients, age, sex/gender, race, type of device, cardiovascular disease severity, and baseline characteristics), and outcomes (e.g., definition, type of outcome, time of assessment, results for outcomes, and any subgroups). In addition, we will utilize three existing frameworks to guide data extraction as they will provide a template to guide data extraction and help to standardize the collection of information across included studies and amongst review authors, with consideration of appropriate levels of detail. This will help to better achieve the overall aim of finding evidence to answer the research questions, while also providing a transparent and reproducible methodology. The frameworks will be used to collect additional information from each included study relevant to the following: descriptions of the intervention and comparator models of follow-up or care (Template for Intervention Description and Replication [TIDieR] checklist [31]), influence of context and setting (Context and Implementation of Complex Interventions [CICI] framework [32]), or equity factors ([PROGRESS-Plus] framework [33, 34]) on the outcomes of interest (Table 2). Detailed data extraction tables are provided in Online Supplement 3.

**Table 2:** Checklist and frameworks for data extraction

**TIDieR checklist**^a^		
1. Brief name: name or phrase describing the intervention. 2. Why: rationale, theory, or goal of elements essential to intervention. 3. What (materials): physical or informational materials used, and where they can be accessed. 4. What (procedure): procedures, activities, and/or processes used in the intervention, including any enabling or support activities. 5. Who provided: background, expertise of provider, and training given. 6. How: modes of delivery, delivered to group or individual. 7. Where: type of location. 8. When and how much: number of times, number of sessions, intensity, and over what time period delivered. 9. Tailoring: what, why, when, and how of planned personalisation/adaptation. 10. Modification: what, why, when, and how of intervention modification during study. 11. How well (planned): if intervention adherence/fidelity was assessed, describe how and by whom, and if any strategies were used to maintain/improve fidelity. 12. How well (actual): if intervention adherence/fidelity was assessed, describe the extent to which the intervention was delivered as planned.		Describing interventions in sufficient detail that they can be replicated and contextual details are documented. Allows for structured accounts of virtual follow-up or care interventions which will facilitate comparisons across included studies and strengthen understanding about how these interventions are designed and delivered.
**CICI checklist**^b, c^		
Context: 7 domains (geographical, epidemiological, socio-cultural, socio-economic, ethical, legal and political). 1. Which aspects of the context interact with the implementation of the intervention? 2. How do these aspects of the context interact with the intervention? 3. How do these aspects of the context interact with implementation? Setting: 4. Which aspects of the setting interact with the intervention? 5. How does the setting interact with the intervention? 6. How does the setting interact with the context? 7. How does the setting interact with the implementation?		Framework used for assessing context and setting (along with implementation) of complex health interventions. We will use as a determinant framework (to conceptualise, describe and understand multiple influences on outcomes).
**PROGRESS-Plus framework**^c^		
PROGRESS: 1. Place of residence 2. Race 3. Occupation 4. Gender 5. Religion 6. Social network and capital 7. Socioeconomic status.	PLUS: 1. Age 2. Disability 3. Sexual orientation 4. Other vulnerable groups.	Permits an equity lens and is used for identifying factors that may affect how disadvantaged groups engage with the intervention, and how PROGRESS-Plus factors may impact access to and use of the intervention. We will also assess the use of equity-relevant outcome measures and summarize results when available to consider whether there are negative or positive impacts to the PROGRESS factors.

^a^Adapted from Hoffmann et al. 2014: Better reporting of interventions: template for intervention description and replication (TIDieR) checklist and guide

^b^Adapted from Pfadenhauer LM, Gerhardus A, Mozygemba K, Lysdahl KB, Booth A, Hofmann B, et al. Making sense of complexity in context and implementation: the context and implementation of complex interventions (CICI) framework. Implement Sci. 2017;12(1):21

^c^CICI items related to implementation activities and intervention description will not be extracted. Intervention descriptions will be extracted using TIDieR checklist items. Implementation experience will be documented in a separate systematic review and qualitative synthesis

^d^Adapted from O’Neill J, Tabish H, Welch V, Petticrew M, Pottie K, Clarke M, et al. Applying an equity lens to interventions: using PROGRESS ensures consideration of socially stratifying factors to illuminate inequities in health. Journal of clinical epidemiology. 2014;67(1):56-64

The TIDieR framework is used to describe interventions in sufficient detail to allow for their replication [31]. Given the heterogeneity of approaches that can be used under the umbrella terms remote monitoring or management, this framework will assist with describing study intervention(s) in detail, and to consider the comparability of interventions described in the individual research studies included. The purpose of the CICI framework is to enable the systematic assessment and documentation of the context, setting, and implementation associated with complex interventions [32]. The purpose of the CICI framework is to enable the systematic assessment and documentation of the context, setting, and implementation associated with complex interventions [32]. This overarching framework outlines potential interacting dimensions of context (including setting) and implementation using eight domains of context (i.e., setting, geographical, epidemiological, socio-cultural, socio-economic, ethical, legal, and political). There are also four implementation domains (i.e., provider, organisation and structure, funding, and policy). Implementation domains will not be a focus for data-extraction in the current review. The PROGRESS framework is used by the Cochrane and Campbell equity group to consider social determinants of heath [34]. PROGRESS stands for place of residence, race/ethnicity/culture/language, occupation, gender/sex, religion, education, socioeconomic status, and social capital. The “Plus” represents added characteristics also associated with potential social disadvantage such as personal characteristics (e.g., age and disability), features of relationships, and time-dependent circumstances (e.g., refugee status) (Table 2).

All data extraction will be completed by one reviewer and checked for completeness and accuracy in its entirety by a second reviewer. Where data are only available from figures (no explicit numerical data are reported) in an included study, we will contact corresponding authors using information listed in the publication to obtain the missing data. Extraction of data for all included studies will be facilitated using forms in Microsoft Excel customized for this systematic review and standardized in advance. Where there are multiple reports for a single study, we will extract data from all reports into one form and document the related citations.

### Quality assessment and risk of bias for individual studies

A risk of bias assessment will be conducted by two independent reviewers, and all disagreements will be resolved through discussion or consultation with a third reviewer as needed. Narrative summaries of all studies included will highlight the strengths and limitations, and tables or figures will be used to present and graphically summarize results. We will assess risk of bias using tools suggested by the Cochrane Collaboration [35], including the Risk of Bias tool [36], and the Risk of Bias in Non-randomized Studies –of Interventions (ROBINS-I) tool [37]. The CLARITY risk of bias tool will be used for case-control studies [38].

### Certainty of evidence

The Grading of Recommendations Assessment, Development, and Evaluation (GRADE) framework will be used to rate the certainty of the evidence of intervention effects [39, 40]. Evidence from all studies will inform the assessment by a single review author. A second independent review author will verify results. A third independent reviewer will adjudicate any differences. Additional GRADE guidance currently under development in an ongoing research project will be used to more specifically contextualize the assessment for considerations relevant to complex health interventions [22]. This approach facilitates incorporation of evidence from the TIDieR, CICI, and PROGRESS-Plus frameworks into the GRADE assessment, and outlines additional considerations relating to context, setting, and other factors that assessors can use in rating the certainty of evidence. Results will be presented in Summary of Findings tables and accompanied with a narrative summary by outcome.

### Analysis

#### Descriptive syntheses

Study characteristics and findings will be summarized descriptively and presented in tabular format. Results from all included studies will be summarized and synthesized narratively.

#### Meta-analyses

Where feasible and appropriate, we will pool the results of included studies using meta-analyses. Feasibility will be based on the availability of sufficient data for each outcome while appropriateness will be assessed through reviewer evaluation of the available data for clinical, methodological, and statistical heterogeneity. Evaluations will be informed through consultation with a biostatistician (statistical and methodological homogeneity) and clinical practitioners (clinical and methodological homogeneity) in cardiology and electrophysiology. Decisions will be made as to the important patient or design factors that may be expected to modify or mediate the outcomes of interest. Results from interventions aimed at monitoring or surveillance of patients and their devices (i.e., virtual follow-up or remote monitoring) will be analyzed separately from interventions where virtual follow-up is combined with virtual therapeutic care (i.e., virtual care or remote patient management).

The results from different study designs will not be pooled. Rather, results will be pooled in separate meta-analyses according to study design. No meta-analyses will be conducted for any study without a control group. Random effects meta-analyses will be performed using the Cochrane Review Manager software (RevMan version 5.3 or the version available at the time). The unit of analysis will be the participant. All dichotomous outcomes (e.g., mortality, stroke) will be pooled using relative risks (randomized, cohort studies, quasi-experimental, or quasi-randomized) or odd ratios (case control studies), and 95% confidence intervals (CIs) will be calculated. Pooling of continuous outcomes will be done using mean differences and corresponding 95% CIs or standardized mean differences where, for example, outcome scales differ. Where adjusted results are reported in effect measures, the analyses will use adjusted results. Differences between unadjusted and adjusted results will be investigated where data are sufficient and discussed. When results are not presented with appropriate measures of variance, the variances will be imputed where possible. If medians or quartiles are reported, estimates of means and associated standard deviations will be calculated [41].

Individual and pooled estimates from all meta-analyses will be presented using tables, and forest plots will be created. Additional visualization approaches may also be used (e.g., harvest or albatross plots [26]). Statistical heterogeneity will be measured and evaluated using forest plots and calculations of Cochran’s chi-square test and the *I*^2^ statistic during meta-analyses, and guidance from the Cochrane Handbook will be used to interpret heterogeneity [35].

#### Subgroup and meta-regression analyses

Subgroup analyses are a straightforward and flexible approach appropriate for evaluating the impact of context, setting, participants, intervention characteristics, and other factor (e.g., equity) influences on outcomes [26]. Where feasible and appropriate, we will use subgroup analyses to explore all of these factors, identifying as many subgroups of interest as possible a priori, and using exploratory analyses to investigate the impact of subgroups identified during the review. The a priori subgroups of interest to be analysed in meta-analyses are as follows:

- Type of device (pacemaker, ICD, CRT);
- Vendor (Medtronic, Abbott, Boston Scientific, Biotronik, LivaNova, or others if listed);
- Severity and type of underlying cardiovascular disease (e.g., disease, New York Heart Association (NYHA) Functional Classification I through IV);
- Remoteness to device clinic (urban, rural, remote);
- Type of residence (e.g., home/personal residence, assisted living residence, long-term care home);
- Length of time using virtual follow-up or care (< 1 year, ≥ 1 to < 2 years, ≥ 2 to < 5 years, 5 years or more);
- Country of delivery;
- Indication for use of virtual follow-up or care (general patient management, safety alert or recall, battery or device replacement imminent).

The impact of important variations in intervention delivery or design identified using the TIDieR checklist will be investigated through subgroup analyses where possible. A priori subgroups of interest related to variation in delivery or design include frequency of transmission (daily, weekly, monthly), follow-up and circle of care (where, by whom), education and training components (by minutes spent educating patient), clinic response to transmissions or alerts, and any key differences identified through TIDieR items. Intervention descriptions will also be used to assess the potential for intervention-generated inequities [28].

All context and setting variables identified using the CICI framework will be subject to subgroup analyses (Table 2). Meta-analyses of the patient, device clinic, and systems levels’ factors will consider impacts to important outcomes at the micro, meso, and macro scales of the context and setting domains [32]. The CICI framework may also capture important aspects of the PROGRESS-Plus factors. All PROGRESS-Plus items will be investigated through subgroup analyses. The credibility of subgroup analyses will be assessed using criteria by Sun et al. using information from each study relevant to the design, analysis, and context (Online Supplement 4) [42]. Where there is a paucity of data to inform analyses based on these factors, they will be considered in the interpretation of the review.

Where the number of studies is sufficient, meta-regression analyses may be used to examine the effects of multiple study features relevant to context, setting, participants, or intervention characteristics, or to explore the mediating effects of intermediate outcomes.

#### Sensitivity analyses

Sensitivity analyses may be considered to evaluate the robustness of pooled estimates using methodological and statistical aspects, including the impact of study design (e.g., randomized controlled trials versus cohort studies versus case-control studies), study quality, date of publication, composition of the study population (i.e., 100% meet eligibility criteria versus “mixed” populations), or varying definitions used for study outcomes.

### Publication bias

Visual tools (e.g., Funnel plots for RCTs) and statistical tests (e.g., Egger’s regression test, Begg’s rank correlation test) will be used to assess publication bias where there are 10 or more included randomized studies [35].

## Reporting

This systematic review will aim to meet all criteria outlined in A Measurement Tool to Assess Systematic Reviews revised tool (AMSTAR 2) [43]. Relevant reporting guidelines will be considered when preparing the written report of systematic review methods and findings (PRISMA [30], PRISMA harms [44], PRISMA equity [45], and the Meta-analysis Of Observational Studies in Epidemiology [MOOSE] checklist [46]). Consideration will also be given to reporting guidance relevant specifically to the TIDieR [31], CICI [32], and PROGRESS-Plus [34] frameworks used in this review.

## Amendments

Any amendments to this protocol will be documented, reflected in edits to the PROSPERO registration, and presented with the findings of the review.

## Dissemination

The results from this systematic review will be published in a peer-reviewed journal and disseminated through various media, including, but not limited to conferences, seminars, congresses, or symposia.

## Discussion

Increases in number of devices implanted combined with an aging population and finite health resource allocations at the system-level may lead to increased reliance on virtual follow-up or care models in the future, while prioritizing consistent, equitable, and timely care as a priority. This systematic review will provide important insight into the potential contextual factors which moderate or mediate the effectiveness, safety, and cost of virtual follow-up or care models for patients with implanted cardiac electronic devices. It will summarize hypothesized variation in studied intervention design and delivery to improve our understanding of this model of follow-up and care, while considering an equity lens to investigate variables that may impact access to and use of healthcare interventions.

Potential threats to this review are the large number of non-RCT studies that may be included, although we believe that their inclusion is necessary to capture important contextual, setting, and other details that may not have been described or of interest in published trials. There is also a possibility that there will be a paucity of quantitative data to answer some of the research questions of interest. Using the TIDieR, CICI and PROGRESS-PLUS frameworks will facilitate descriptive synthesis in this case, and gaps in knowledge to answer any of the current research questions will provide a foundation for additional quantitative and qualitative primary research.

Considerations for context, setting, and equity are necessary given the complex nature of virtual follow-up and care, and the reciprocal complexity of the health care system in which it is used. Results will be used to identify factors in individuals, intervention design, delivery, or potential implementation contexts or settings leading to success in terms of outcomes for the patient. The findings will inform future work that will aim to improve access, uptake, use, and spread of virtual follow-up and care models for CIED patients that will be applicable to inform improvements in post-implant care in both high or low and middle income countries. Ideally, new practice guidelines for management of this population will be developed that adequately consider all evidence relevant to the delivery of context and equity-sensitive patient-centred arrhythmia management strategies for virtual follow-up and care.

### Provenance and peer review

Not commissioned; externally peer reviewed.

## Supplementary information

**Additional file 1.** PRISMA-P checklist.

**Additional file 2.** Proposed search strategy.

**Additional file 3.** Proposed data extraction form.

**Additional file 4.** Subgroup analyses criteria.

## Data Availability

Not applicable

## Abbreviations

AMSTAR 2: Assessing the Methodological Quality of Systematic Reviews (version 2)
CICI: Context and Implementation of Complex Interventions
CI: Confidence interval
CIED: Cardiac implantable electronic device
CRT: Cardiac resynchronization therapy
GRADE: Grading of Recommendations, Assessment, Development and Evaluations
ICD: Implantable cardioverter defibrillator
MOOSE: Meta-analyses of Observational Studies in Epidemiology
PRISMA: Preferred Reporting Items for Systematic Reviews and Meta-Analyses
PROGRESS-Plus: Place of residence, race/ethnicity, occupation, gender, religion/culture, education, socio-economic status, social capital/networks, plus other important factors which impact on health equity
RCT: Randomized controlled trial
TIDieR: Template for Intervention Description and Replication
