# Supplementary material for: Virtual follow-up and care for patients with cardiac electronic implantable devices: protocol for a systematic review
Source: Syst Rev. 2020 Jun 27;9:153. doi: 10.1186/s13643-020-01406-6 (PMC7321546; doi:10.1186/s13643-020-01406-6)
Supplement: Supplementary file 3 — Additional file 3. Proposed data extraction form. [file 13643_2020_1406_MOESM3_ESM.docx]

| ADDITIONAL FILE 3. Proposed data extraction form for included studies. |
| --- |
| STUDY CHARACTERISTICS |
| \| **Ref ID:** \|  \| \| --- \| --- \| \| **Author(s):** \|  \| \| **Publication Year:** \|  \| \| **Publication Title:** \|  \| \| **Country** (where the study was conducted, or the country of the corresponding author’s affiliation): \|  \| \| **Funding sources:** \|  \| \| **Study is relevant for clinical research question(s):** \| 1, 2, 3, 4, 5, 6, 7, 8, 9, 10 *(checkboxes)* \| \| **Study reported as abstract only** \|  \| \| **Study reported as study registry record only** \|  \| \| **Language of publication** (if abstract and/or full-text non-English) \|  \| |
| METHODOLOGY |
| \| **Study design:** \| Secondary research:  HTA or SR  Primary research:  RCT, non-randomized controlled trial, quasi-randomized controlled trial, cohort study, case-control study *(checkboxes)* \| \| --- \| --- \| \| **Study objectives:** \|  \| \| **Inclusion criteria:** \|  \| \| **Exclusion criteria:** \|  \| \| **Recruitment period:** \|  \| \| **Description of primary and secondary outcomes reported:** \|  \| \| **Description of subgroup analyses of interest:** \|  \| \| **Hypothesis:** \| Superiority/non-inferiority/other *(checkboxes)* \| |
| POPULATION - Overall |
| \| **Number of participants consented:** \|  \| \| --- \| --- \| \| **Number of participants randomized:** \|  \| \| **Age:** \|  \| \| **Sex** (Female/Male): \|  \| \| **Race:** \|  \| \| **BASELINE CHARACTERISTICS** \|  \| \| **Type of device:** \| ICD, CRT, PM *(checkboxes)* \| \| **Indication for device implant:** \| Primary, secondary, other *(checkboxes)* \| \| **Device-naïve** (first implant**) or device-experienced** (change of device, battery change, lead change)? \|  \| \| **Lives in rural, urban, remote** \|  \| \| **LVEF <= 35%** \|  \| \| **NYHA Class (I,II,III,IV)** \|  \| \| **Device vendor** (e.g., Medtronik, Biotronik, etc.) \| Name/extract all if mixed (with proportions) \| \| **Cardiac medications** \|  \| \| **Relevant co-morbidities reported by study author(s)** \|  \| \| **Pacemaker dependency:** \|  \| \|  \|  \| |
| POPULATION – Intervention Group |
| \| **Number of participants consented:** \|  \| \| --- \| --- \| \| **Number of participants randomized:** \|  \| \| **Age:** \|  \| \| **Sex** (Female/Male): \|  \| \| **Race:** \|  \| \| **BASELINE CHARACTERISTICS** \|  \| \| **Type of device:** \| ICD, CRT, PM *(checkboxes)* \| \| **Indication for device implant:** \| Primary, secondary, other *(checkboxes)* \| \| **Device-naïve (**first implant) **or device-experienced** (change of device, battery change, lead change)? \|  \| \| **Lives in rural, urban, remote** \|  \| \| **LVEF <= 35%** \|  \| \| **NYHA Class (I,II,III,IV)** \|  \| \| **Device vendor (**e.g., Medtronik, Biotronik, etc.) \| Name/extract all if mixed (with proportions) \| \| **Cardiac medications** \|  \| \| **Relevant co-morbidities reported by study author(s)** \|  \| \| **Pacemaker dependency:** \|  \| \|  \|  \| |
| POPULATION – Comparator Group |
| \| **Number of participants consented:** \|  \| \| --- \| --- \| \| **Number of participants randomized:** \|  \| \| **Age:** \|  \| \| **Sex (Female/Male):** \|  \| \| **Race:** \|  \| \| **BASELINE CHARACTERISTICS** \|  \| \| **Type of device:** \| ICD, CRT, PM *(checkboxes)* \| \| **Indication for device implant:** \| Primary, secondary, other *(checkboxes)* \| \| **Device-naïve** (first implant) **or device-experienced** (change of device, battery change, lead change)? \|  \| \| **Lives in rural, urban, remote** \|  \| \| **LVEF <= 35%** \|  \| \| **NYHA Class (I,II,III,IV)** \|  \| \| **Device vendor (**e.g., Medtronik, Biotronik, etc.) \| Name/extract all if mixed (with proportions) \| \| **Cardiac medications** \|  \| \| **Relevant co-morbidities reported by study author(s)** \|  \| \| **Pacemaker dependency:** \|  \| \|  \|  \| |
| INTERVENTION and COMPARISON (TIDieR checklist^a^) |
| \|  \| BRIEF NAME \|  \| \| --- \| --- \| --- \| \| 1. \| Provide the name or a phrase that describes the intervention. \|  \| \|  \| **WHY** \|  \| \| 2. \| Describe any rationale, theory, or goal of the elements essential to the intervention. \|  \| \|  \| **WHAT** \|  \| \| 3. \| Materials: Describe any physical or informational materials used in the intervention, including those provided to participants or used in intervention delivery or in training of intervention providers. Provide information on where the materials can be accessed (e.g. online appendix, URL). \|  \| \| 4. \| Procedures: Describe each of the procedures, activities, and/or processes used in the intervention, including any enabling or support activities. \|  \| \|  \| **WHO PROVIDED** \|  \| \| 5. \| For each category of intervention provider (e.g. psychologist, nursing assistant), describe their expertise, background and any specific training given. \|  \| \|  \| **HOW** \|  \| \| 6. \| Describe the modes of delivery (e.g. face-to-face or by some other mechanism, such as internet or telephone) of the intervention and whether it was provided individually or in a group. \|  \| \|  \| **WHERE** \|  \| \| 7. \| Describe the type(s) of location(s) where the intervention occurred, including any necessary infrastructure or relevant features. \|  \| \|  \| **WHEN and HOW MUCH** \|  \| \| 8. \| Describe the number of times the intervention was delivered and over what period of time including the number of sessions, their schedule, and their duration, intensity or dose. \|  \| \|  \| **TAILORING** \|  \| \| 9. \| If the intervention was planned to be personalised, titrated or adapted, then describe what, why, when, and how. \|  \| \|  \| **MODIFICATIONS** \|  \| \| 10. \| If the intervention was modified during the course of the study, describe the changes (what, why, when, and how). \|  \| \|  \| **HOW WELL** \|  \| \| 11. \| Planned: If intervention adherence or fidelity was assessed, describe how and by whom, and if any strategies were used to maintain or improve fidelity, describe them. \|  \| \| 12. \| Actual: If intervention adherence or fidelity was assessed, describe the extent to which the intervention was delivered as planned. \|  \| |
| REPORTED OUTCOMES |
| \| Primary (including definition) \|  \| \| --- \| --- \| \| Secondary (including definition) \|  \| \| Length of follow-up \|  \| \| Loss to follow-up (by group) \|  \| |
| RESULTS (to be completed for each follow-up time reported, for each outcome reported) |
| \| **OUTCOME** \|  \|  \| \| --- \| --- \| --- \| \| Follow-up time point: ____________________________ \| **Intervention Group** \| **Comparison Group (s)** \| \| Outcome: _____________________________________ \|  \|  \| \| Absolute number (events/persons with outcome) \|  \|  \| \| Change in outcome from baseline \|  \|  \| \| % change in outcome from baseline \|  \|  \| \| % of patients with outcome, or outcome (>,<) threshold [specify threshold] \|  \|  \| \| Outcome specifics: Definitions if required, scale, tool or instrument used, other a necessary \|  \|  \| \| **ADVERSE EVENT OR COMPLICATION** \|  \|  \| \| Specify type, n, and % \|  \|  \| |
| CICI checklist^b, c^ |
| CONTEXT: *7 domains (geographical, epidemiological, socio-cultural, socio-economic, ethical, legal and political).*   1. Which aspects of the context interact with the implementation of the intervention? 2. How do these aspects of the context interact with the intervention? 3. How do these aspects of the context interact with implementation?  \| **CONTEXT:** \| \| **Differences by outcome noted by domain** \| \| \| --- \| --- \| --- \| --- \| \| **Intervention Group** \| **Comparison Group (s)** \| \| Geographical \| - Geography (e.g. altitude, desert, forest, water) - Climate (e.g. temperature, rainfall) - Human land use (e.g. degree of urbanisation, agriculture, industry) - Infrastructure (e.g. water and sanitation, energy, transport) - Access to health care system - Geographical isolation   Relevant changes over time (e.g. infrastructure development, crop failures) \|  \|  \| \| Epidemiological \| - Demographics (life expectancy, gender, age, ethnicity, genetic factors) - Population density, fertility patterns, family size - Incidence/prevalence and severity of disease, morbidity and mortality - Spatial distribution of disease across geographical areas   Relevant changes over time (e.g. epidemics) \|  \|  \| \| Socio-economic \| - Social or socio-economic status attributed to education, income, occupation marriage, or gender - Financial aspects (income, wealth) - Occupational aspects (employment status, working conditions) - Living conditions (housing, neighbourhood characteristics) - Determinants of needs of people directly affected by disease/condition - Burden of disease - Determinants of needs of people indirectly affected by disease/condition - Fiscal environment - Market environment - Access to health care system   Relevant changes over time (e.g. inflation, recession, economic crisis) \|  \|  \| \| Socio-cultural \| - Language and means of communication - Symbols, heroes, rituals - Values (e.g. evil vs. good, dirty vs. clean, dangerous vs. safe, abnormal vs. normal) - Beliefs (e.g. superstition, fate or destiny) - Religiosity and spirituality - Knowledge and perceptions (e.g. with respect to significance of health issue, options for resolving health issue, multiple benefits and drawbacks of technology) - Lifestyle (population’s patterns in nutrition, smoking, substance abuse) - Discrimination - Social capital and resources available through social relationships, specifically social networks, norms of reciprocity, and trust - Social cohesion, including relational, material, and political dimensions, information exchange, networks of support, and informal social control - Historical and contemporary social power relations - Sociodemographic profiles - Psychosocial factors - Social and societal context - Structural social inequalities (e.g. Gender inequalities, caste system) - Community characteristics and level of coordination/involvement with community   Relevant changes over time (e.g. social changes or social movements) \|  \|  \| \| Political \| - Political system and civil society’s structure - Players, interests, resources, objectives, formal and informal rules - Distribution of power - Political culture and socio-political climate - State-society relations - Political situation including political stability and absence of violence, government effectiveness, voice and accountability, control of corruption, rule of law, regulatory quality, participation, accountability, transparency, efficiency, decency, and fairness - Economic management, economic policy and political framework of markets - Politics and gender - International integration - Ideology - Short-term thinking - Influential people - Payer, donor or funder policies - Political authority - Health Care System (e.g. governance and leadership, resources, service delivery, integration of patient’s needs and perspective) - Access to health care system   Relevant changes over time (e.g. political reform*, change of government) \|  \|  \| \| Legal \| - Norms, values and beliefs underlying legislation - Specific legislation (e.g. patient rights, data protection) - Regulatory provisions concerning healthcare personnel and their rights and duties - Guidelines - Decision-making in care delivery - Sharing of information with indirectly affected stakeholders - Legislation   Relevant changes over time (e.g. introduction of new regulation or legislation) \|  \|  \| \| Ethical \| - Refinement according to applications: - Autonomy - Moral distress - Privacy - Conflicting interests - Morality and beliefs - Ethical principles and code of conduct   Relevant changes over time \|  \|  \|   SETTING:   1. Which aspects of setting were reported in the study? 2. Which aspects of the setting interact with the intervention? 3. How does the setting interact with the intervention? 4. How does the setting interact with the context? 5. How does the setting interact with the implementation?  \| **SETTING:** \| \| **EXTRACTED DATA:** \| \| \| --- \| --- \| --- \| --- \| \| **Intervention Group** \| **Comparison Group (s)** \| \| Setting \| • City, region, country (e.g. urban, rural)  • Type of study site (e.g. primary care, hospital, home, school, occupational setting  • Number of study sites  • Physical characteristics  • Work environment  • Effect of location on affected stakeholder  • Relevant changes over time (e.g. urbanisation) \| *Includes both clinical and participant setting.* \| *Includes both clinical and participant setting.* \| |
| PROGRESS-Plus Framework^d^ |
| \| **OUTCOME** \|  \|  \| \| --- \| --- \| --- \| \| Follow-up time point: ____________________________ \|  \|  \| \| Outcome: _____________________________________ \|  \|  \| \| **PROGRESS:** \| **Intervention Group** \| **Comparison Group (s)** \| \| PLACE OF RESIDENCE \|  \|  \| \| Different baseline risk \|  \|  \| \| Different relative effects \|  \|  \| \| Different absolute effects \|  \|  \| \| Different certainty of evidence \|  \|  \| \| RACE \|  \|  \| \| Different baseline risk \|  \|  \| \| Different relative effects \|  \|  \| \| Different absolute effects \|  \|  \| \| Different certainty of evidence \|  \|  \| \| OCCUPATION \|  \|  \| \| Different baseline risk \|  \|  \| \| Different relative effects \|  \|  \| \| Different absolute effects \|  \|  \| \| Different certainty of evidence \|  \|  \| \| GENDER \|  \|  \| \| Different baseline risk \|  \|  \| \| Different relative effects \|  \|  \| \| Different absolute effects \|  \|  \| \| Different certainty of evidence \|  \|  \| \| RELIGION \|  \|  \| \| Different baseline risk \|  \|  \| \| Different relative effects \|  \|  \| \| Different absolute effects \|  \|  \| \| Different certainty of evidence \|  \|  \| \| SOCIAL NETWORK AND CAPITAL \|  \|  \| \| Different baseline risk \|  \|  \| \| Different relative effects \|  \|  \| \| Different absolute effects \|  \|  \| \| Different certainty of evidence \|  \|  \| \| SOCIOECONOMIC STATUS. \|  \|  \| \| Different baseline risk \|  \|  \| \| Different relative effects \|  \|  \| \| Different absolute effects \|  \|  \| \| Different certainty of evidence \|  \|  \| \| **PLUS:** \|  \|  \| \| AGE \|  \|  \| \| Different baseline risk \|  \|  \| \| Different relative effects \|  \|  \| \| Different absolute effects \|  \|  \| \| Different certainty of evidence \|  \|  \| \| DISABILITY \|  \|  \| \| Different baseline risk \|  \|  \| \| Different relative effects \|  \|  \| \| Different absolute effects \|  \|  \| \| Different certainty of evidence \|  \|  \| \| SEXUAL ORIENTATION \|  \|  \| \| Different baseline risk \|  \|  \| \| Different relative effects \|  \|  \| \| Different absolute effects \|  \|  \| \| Different certainty of evidence \|  \|  \| \| OTHER VULNERABILE GROUP [state group] \|  \|  \| \| Different baseline risk \|  \|  \| \| Different relative effects \|  \|  \| \| Different absolute effects \|  \|  \| \| Different certainty of evidence \|  \|  \| |

HTA=health technology assessment; SR=systematic review; RCT=randomized controlled trial; n=number

a . Adapted from Hoffmann TC, Glasziou PP, Boutron I, Milne R, Perera R, Moher D, et al. Better reporting of interventions: template for intervention description and replication (TIDieR) checklist and guide. BMJ. 2014;348:g1687.

b .Adapted from Pfadenhauer LM, Gerhardus A, Mozygemba K, Lysdahl KB, Booth A, Hofmann B, et al. Making sense of complexity in context and implementation: the Context and Implementation of Complex Interventions (CICI) framework. Implement Sci. 2017;12(1):21.

c .CICI items related to implementation activities and intervention description will not be extracted. Intervention descriptions will be extracted using TIDieR checklist items.

d Adapted from O'Neill J, Tabish H, Welch V, Petticrew M, Pottie K, Clarke M, et al. Applying an equity lens to interventions: using PROGRESS ensures consideration of socially stratifying factors to illuminate inequities in health. Journal of clinical epidemiology. 2014;67(1):56-64.
