# Supplementary material for: Virtual follow-up and care for patients with cardiac electronic implantable devices: protocol for a systematic review
Source: Syst Rev. 2020 Jun 27;9:153. doi: 10.1186/s13643-020-01406-6 (PMC7321546; doi:10.1186/s13643-020-01406-6)
Supplement: Supplementary file 4 — Additional file 4. Subgroup analyses criteria. [file 13643_2020_1406_MOESM4_ESM.pdf]

ADDITIONAL FILE 4. Criteria to assess the credibility of subgroup analyses (Sun et al, 2010)(1).

**DESIGN**	
1.	Is the subgroup variable a characteristic measured at baseline or after randomisation?
2.	Is the effect suggested by comparisons within rather than between studies?
3.	Was the hypothesis specified a priori?
4.	Was the direction of the subgroup effect specified a priori?
5.	Was the subgroup effect one of a small number of hypothesised effects tested?
**ANALYSIS**	
6.	Does the interaction test suggest a low likelihood that chance explains the apparent subgroup effect?
7.	Is the significant subgroup effect independent?
**CONTEXT**	
8.	Is the size of the subgroup effect large?
9.	Is the interaction consistent across studies?
10.	Is the interaction consistent across closely related outcomes within the study?
11.	Is there indirect evidence that supports the hypothesised interaction (biological rationale)?

1. Sun X, Briel M, Walter SD, Guyatt GH. Is a subgroup effect believable? Updating criteria to evaluate the credibility of subgroup analyses. BMJ. 2010;340:c117.
